# Deep Learning-Based Transfer Learning for Classification of Skin Cancer

**DOI:** 10.3390/s21238142

**Published:** 2021-12-06

**Authors:** Satin Jain, Udit Singhania, Balakrushna Tripathy, Emad Abouel Nasr, Mohamed K. Aboudaif, Ali K. Kamrani

**Affiliations:** 1 Department of Information Technology and Engineering, Vellore Institute of Technology, Vellore 632014, Tamil Nadu, India; satinsunil.jain2017@vitstudent.ac.in (S.J.); tripathybk@vit.ac.in (B.T.); 2 Department of Computer Science and Engineering, Vellore Institute of Technology, Vellore 632014, Tamil Nadu, India; udit.singhania2017@vitstudent.ac.in; 3 Industrial Engineering Department, College of Engineering, King Saud University, Riyadh 11421, Saudi Arabia; maboudaif@ksu.edu.sa; 4 Industrial Engineering Department, College of Engineering, University of Houston, Houston, TX 77204-4008, USA; akamrani@uh.edu

**Keywords:** image classification, skin lesion, CNN, transfer learning, artificial intelligence

## Abstract

One of the major health concerns for human society is skin cancer. When the pigments producing skin color turn carcinogenic, this disease gets contracted. A skin cancer diagnosis is a challenging process for dermatologists as many skin cancer pigments may appear similar in appearance. Hence, early detection of lesions (which form the base of skin cancer) is definitely critical and useful to completely cure the patients suffering from skin cancer. Significant progress has been made in developing automated tools for the diagnosis of skin cancer to assist dermatologists. The worldwide acceptance of artificial intelligence-supported tools has permitted usage of the enormous collection of images of lesions and benevolent sores approved by histopathology. This paper performs a comparative analysis of six different transfer learning nets for multi-class skin cancer classification by taking the HAM10000 dataset. We used replication of images of classes with low frequencies to counter the imbalance in the dataset. The transfer learning nets that were used in the analysis were VGG19, InceptionV3, InceptionResNetV2, ResNet50, Xception, and MobileNet. Results demonstrate that replication is suitable for this task, achieving high classification accuracies and F-measures with lower false negatives. It is inferred that Xception Net outperforms the rest of the transfer learning nets used for the study, with an accuracy of 90.48. It also has the highest recall, precision, and F-Measure values.

## 1. Introduction

Skin cancer is one of the most common cancers worldwide. It greatly affects the quality of life. The most common cause is the over exposure of skin to ultraviolet radiations coming from the sun [1]. The rate of being affected when exposed to UV radiations is higher in fair skinned, more sun-sensitive people than in dark skinned, less sun-sensitive people [2].

Invasive melanoma represents about 1% of all skin cancer cases, but it contributes to the majority of deaths in skin cancer. Incidence of melanoma skin cancer has risen rapidly over the past 30 years. It is estimated that in 2021, 100,350 new cases of melanoma will be diagnosed in the US and around 6850 people will eventually die from it [3].

The best way to control skin cancer is its early detection and prevention [4]. Awareness of new or changing skin spots or growths, particularly those that look unusual, should be evaluated. Any new lesions, or progressive change in a lesion’s appearance (size, shape, or color), should be evaluated by a clinician. With the advent of deep learning concepts [5], we can classify skin cancer detection in seven diagnostic categories, namely melanocytic nevi, melanoma, benign keratosis-like lesions, basal cell carcinoma, actinic keratosis, vascular lesions, and dermatofibroma. Generally, a dermatologist specializing in skin cancer detection follows a fixed sequence, i.e., starting with a visual examination of the suspected lesion with naked eyes, followed by a dermoscopy and finally a biopsy [6].

In today’s era, with the usage of artificial intelligence and deep learning [7] in medical diagnostics [8], the efficiency of predicting a result increases exponentially as compared to the dependency on a visual diagnostic [9,10,11], Machine learning also has applications in many other fields, alongside the medical field [12,13,14,15,16]. The convolutional neural network (CNN) is an important artificial intelligence algorithm in feature selection and object classification [17,18,19]. Deep convolutional neural networks (DCNN) help in classifying skin lesions into seven different categories, with the help of their dermoscopic images, covering all the lesions found in skin cancer identification. Although DNNs require a large amount of data for training [20,21], they have an appealing impact on medical image classification [22,23]. DNNs train a network of large-scale datasets using high performance GPUs, thus providing a better outcome [17,24]. Deep learning algorithms backed by these high performing GPUs in computing large datasets have shown better performance than humans in skin cancer identification [25].

### Literature Background

Deep learning gained popularity during the last decade [26,27,28]. Convolutional neural networks have been widely used in the classification of diseases [29,30]. It is challenging to train a CNN architecture if the datasets have a limited number of training samples. In [18], a partial transferable CNN was proposed in order to cope with a new dataset with a different spatial resolution, a different number of bands, and variation in the signal-to-noise ratio. The experimental results using different state-of-the-art models show that partial CNN transfer with even-numbered layers provides better mapping accuracy for the target dataset with a limited number of training samples. In [19], a novel method using transfer learning to deal with multi-resolution images from various sensors via CNN is proposed. CNN trained for a typical image data set, and the trained weights were transferred to other data sets of different resolutions. Initially, skin cancer diseases were divided only into two categories, benign or malignant. Canziani et al. [31] made use of machine learning algorithms, such as K-Means and SVM, and achieved an accuracy of 90%. Codella [32] makes use of the ISIC 2017 dataset, which consists of three categories of skin cancer, with conventional machine learning methods, in order to predict melanoma precisely but suffered from inaccurate results due to dataset bias and incomplete dermoscopic feature annotations. Another case of skin lesion classification [33] on the same dataset, in which a proposed lesion indexing network (LIN) was introduced, managed to attain the 91.2% area under the curve. However, it was performed on ISIC 2017, and no work has been recreated on ISIC 2018. There are also some datasets that divide the skin lesion into 12 different categories. Han [34] used the Asan dataset, med-node dataset, and atlas site images, which, together, consisted of 19,398 images divided into 12 categories. He used Resnet architecture for classification and achieved an accuracy of 83%. His paper was moreover inclined towards proving that the proposed dataset was better than those taken in comparison. Chaturvedi et al. [35] made use of the HAM10000 dataset, seven different types of skin lesion, using MobileNet in skin lesion detection, and achieved an accuracy of 83%. Milton [36] presented with transfer learning algorithms that were trained on the HAM10000 dataset and used fine-tuning and freezing of two epochs. Here, PNASNet-5-Large was used, which gave an accuracy of 76%. HAM10000 being an unbalanced dataset with a large difference in total images for each class makes it harder to generalize the features of the lesions. Nugroho [25] made his own custom CNN model, which produced 78% accuracy on the HAM10000 dataset. Kadampur [5] introduced an online method without coding for the classification of HAM10000 diseases and training on the cloud. Although the advantage of the above-mentioned research works is that they provide a straightforward algorithm approach and acceptable accuracy, most of them did not consider all types of legions and used relatively old datasets.

It was found that most papers had done classification of lesions [37] in the three standard categories, i.e., basal cell carcinoma, squamous cell carcinoma, and melanoma. The dataset used for classification was not so recent and not sufficient enough to identify all types of lesions [38]. By keeping all this in mind, three objectives were framed

- To classify the images from HAM10000 dataset into seven different types of skin cancer.
- To use transfer learning nets for feature selection and classification so as to identify all types of lesions found in skin cancer.
- To properly balance the dataset using replication on only training data and perform a detailed analysis using different transfer learning models.

In this paper, HAM10000 dataset was used to train the model for skin cancer classification. All the six transfer learning nets were compared, and their training and validation loss, training and validation accuracy, along with their individual confusion matrices, were plotted. A comparative analysis of accuracy was then performed for all these learning nets and concluded with the model, which gave the highest accuracy in identifying all the lesions.

## 2. Materials and Methods

### 2.1. Dataset Description for Skin Lesion

**Figure 1:**
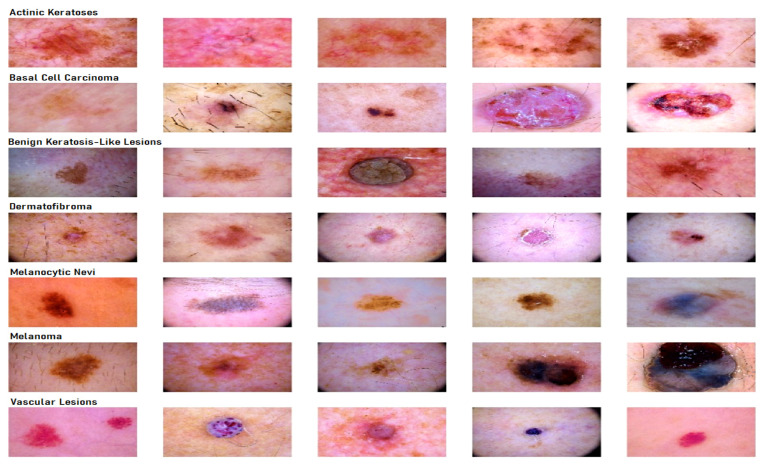
Seven different types of diseases caused from lesions.

**Figure 2:**
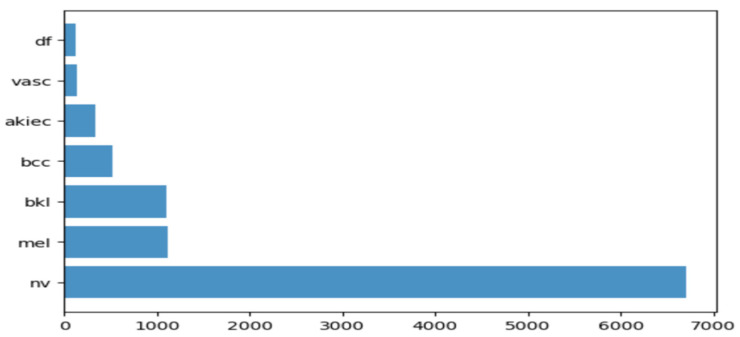
Occurrence of images of each type of skin cancer.

To carry out the research work, we used HAM10000 dataset (human against machine) [39], which has 10015 dermatoscopic images and seven different classes, such as actinic keratosis (akiec) (327), basal cell carcinoma (bcc) (541), benign keratosis (bkl) (1099), dermatofibroma (df) (155), melanocytic nevi (nv) (6705), melanoma (mel) (1113), and vascular skin lesions (vasc) (142). Seven types of lesions [29] are shown in Figure 1, along with their occurrences in Figure 2, where x-axis represents the type of lesion and y-axis represents the corresponding count. The same dataset was divided into training, testing, and validation sets, so that there was no discrepancy in the results.

### 2.2. Transfer Learning Nets

In this section, the focus is on transfer learning and the models used in the research are discussed briefly [40]. Transfer learning is a machine learning method in which a model developed from one task is reused in another. It is generally used when we do not have enough training data. However, the data issue can be solved with data augmentation. The main reason why we need transfer learning is because Melanoma and benign lesions have high similarity, so it takes a long time to identify and classify them. Furthermore, transfer learning is more efficient in classifying between similar lesions, making it a first choice. Transfer learning nets are trained on large datasets and their model weights are frozen, and the last few layers are changed for a different dataset. In this paper, the models we used for comparison were VGG19, InceptionV3, InceptionResNetV2, Resnet50, Xception. and MobileNet. However, here, we not only used the frozen weights, but we also retrained them on our dataset so that the network layers had better precision in distinguishing between seven different types of lesions. We trained the models on the skin lesion dataset using these six transfer learning nets and analyzed their predictions. In addition to this, we plotted their training and validation loss, training and validation accuracy, along with their individual confusion matrices. A comparative analysis of accuracy of all these learning nets was then performed, and we determined the net that gave the highest accuracy in identifying all the lesions.

#### 2.2.1. VGG19

This network is characterized by its simplicity. It has five blocks each of 3 × 3 convolutional layers stacked on top of each other. Volume size is reduced by max pooling of 2 × 2 kernels and a stride of 2. It is followed by two fully-connected layers, each of 4096 nodes, with ReLU activation function. The final layer has 1000 nodes with softmax as its activation function [41]. VGG19 has about 143 million parameters in total. Some applications of VGG net are mentioned by Canziani [31] in his paper.

#### 2.2.2. InceptionV3

InceptionV3 [42] is the refined version of the GoogLeNet architecture [43]. The basic idea of this net is to make this process simpler and more efficient. The Inception module acts as a multi-level feature extractor. It computes 1 × 1, 3 × 3, and 5 × 5 convolutions within the same module of the network. The outputs of these filters are then stacked on each other and fed into the next layer in the network.

#### 2.2.3. InceptionResnetv2

In this net [44], the residual version of Inception nets is used rather than simple inception modules. Each Inception block is followed by a filter-expansion layer (1 × 1 convolution without activation), which is used for scaling up the dimensionality of the filter bank before the addition to match the depth of the input. Inception-ResNet-v2 matches the computational cost of the Inception-v4 network. The difference between residual and non-residual Inception variants is that in the case of Inception-ResNetv2; batch normalization is used only on top of the traditional layers, but not on top of the summations.

#### 2.2.4. ResNet50

These are the deeper convolutional neural nets, which make use of skip connections [45]. These residual blocks greatly resolve gradient degradation and also reduce total parameters. Residual Networks (ResNet [46]) architecture follows two simple design rules. Firstly, for the same output map size, layers have the same number of filters, and secondly, when the feature map size is halved, the filters count is doubled. Batch normalization is performed after each convolution layer and before ReLU activation function. If the input and output have the same size, the shortcut is used. When there is an increase in dimensions, the projection shortcut is used. 

#### 2.2.5. Xception

The Xception [47] architecture is an extension of the Inception architecture. It replaces the standard Inception modules with depth wise separable convolutions. It does not perform partitioning on input data and maps the spatial correlations for each output channel separately. The Xception net then performs 1 × 1 depth wise convolution, which captures cross-channel correlation. It slightly outperforms Inception V3 in terms of smaller data and vastly on bigger data.

#### 2.2.6. MobileNet

This net makes use of depth wise separable connections, similar to the Xception net. For MobileNets [48], the depth wise convolution applies a single filter to each input channel. The pointwise convolution then applies a 1 × 1 convolution to combine the outputs of the depth wise convolution. A standard convolution layer does both: filters and combines inputs into a new set of outputs in one step. The depth wise separable convolution splits this into two layers: a separate layer for filtering and a separate layer for combining. This factorization has the effect of drastically reducing computation and model size. MobileNet is particularly useful for mobile and embedded vision applications. It has less parameters compared to others and also less complexity. This architecture is a concise form of the Xception and Inception nets.

### 2.3. Proposed Methodology

**Figure 3:**
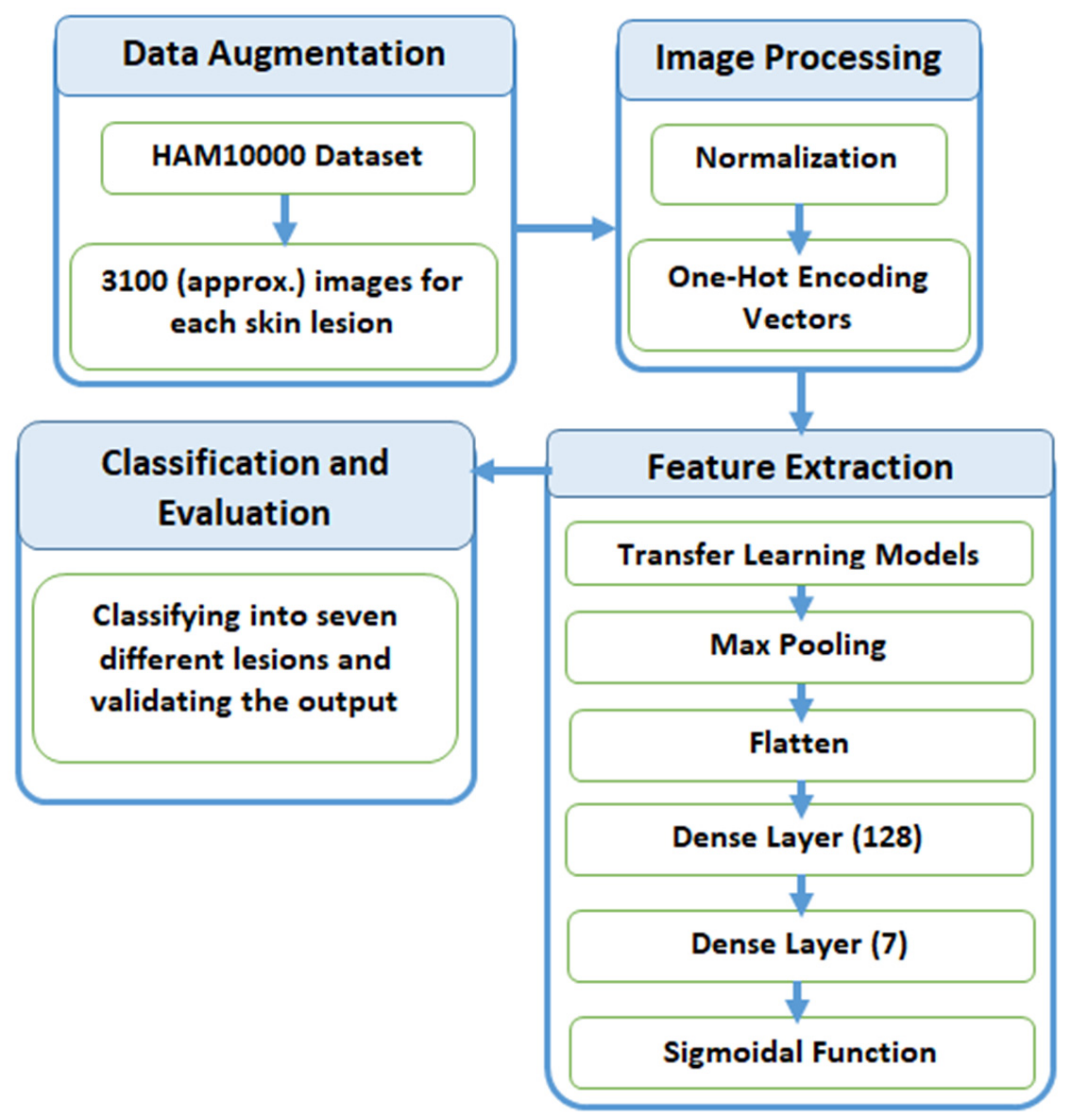
Process flow diagram of the proposed method.

In this section, we explain the process of classification of skin lesions. The main issue with the dataset is that it is highly imbalanced and contains a lot of duplicated images. Therefore, we made use of data augmentation to resolve this issue. Figure 3 provides a diagrammatic representation of the proposed method.

#### 2.3.1. Data Augmentation

When inspecting the dataset, it was observed that a lot of images were just a replication of each other, which is not beneficial for our models. We identified the unique images in the dataset, which amounted to be around 5514. We split these images into training and testing data of 80% and 20%, respectively. Training data was further divided into 90% training and 10% validation. Training data had approximately 4000 images, which was very few, and also, the classes were unbalanced because a few classes had many more images compared to others. After removing duplicates, Melanocytic Nevi had 3179 images and Dermatofibroma had only 28 images. We tackled this problem by replicating the class with low data by multiplying it by a factor that would produce data close to the class with the highest data.

Table 1 explains the frequency of images of each label before and after augmentation. All the images were multiplied by a factor k so that they could lie closest to Melanocytic Nevi. The above technique was used to avoid the problem of class imbalance. Here, we expanded the training dataset artificially. We altered the training data with small transformations to reproduce variations. A few techniques, such as rotation, zooming, and shifting vertically and horizontally, were implemented. All the images were part of the HAM10000 dataset, and their dimensions were resized from 450 × 600 to 128 × 128 dimensions for convenience in processing.

After training the model with the initial training dataset, it was observed that even though a good accuracy was obtained, an observation of the classification matrix reflected the real picture. One class (Melanocytic Nevi) was classified a majority of the time, since it had the highest frequency. This indicated that the model was biased and was not able to predict or classify other low frequency classes. To overcome this situation, it was required to equalize the distribution of classes and let the model understand each class. The dataset was augmented by increasing the frequency of each class so that all the classes had the same number of images. As a result, a better performing wholesome model could be realized.

#### 2.3.2. Preprocessing

After the image acquisition task, we performed image preprocessing. We had three channels of data corresponding to the colors Red, Green, and Blue (RGB). Pixel levels are usually [0–255]. Image preprocessing involves normalization of the images. In normalization, mean and standard deviation of all images in the dataset is calculated. The mean of all the images was subtracted from initial images and then the obtained result was divided by standard deviation. On the other hand, the seven diseases were one, hot encoded, i.e., a binary column for each category was created.

Image width, image height = 128, 128

3, channels = pixel levels in the range [0–255]

(1) $$\mathrm{Normalization}=\frac{x-\mu }{\sigma }$$

where *x* is the original feature vector, μ is the mean, and σ is the standard deviation.

#### 2.3.3. Feature Extraction

Feature extraction is the most crucial step in classification. Feature extraction was carried out by pre-trained transfer learning models. This involves looking up important features in an image and then deriving information from them. Several CNNs are stacked up back-to-back in order to make a model.

Here, we used pre-trained models, such as VGG19, InceptionV3, Resnet50, Xception, InceptionResNetV2, and MobileNet. All of the above pre-trained nets used the weights of the Imagenet. The bottom layers were Max Pooling, which calculates the maximum value of each patch of feature map, Flatten, which converts the 3d array into a 1d array, Dense layer with 128 neutrons and finally a Dense layer with seven neurons, corresponding to seven different diseases with sigmoidal activation function.

#### 2.3.4. Classification and Evaluation

The final layer outputs an array of seven values, which indicates the probability of each category of disease. The class number was in correspondence to seven different skin cancers. The class numbers assigned for different lesions were actinic keratosis (0), basal cell carcinoma (1), benign keratosis like lesions (2), dermatofibroma (3), melanocytic nevi (4), melanoma (5), and vascular skin lesions (6), and in the evaluation phase, we used a validation dataset for validating the different nets for the skin lesion dataset.

## 3. Results

In this section, the experimental results and analysis of our models used on the HAM10000 dataset are presented. The results of six different types of transfer learning models, VGG19, InceptionV3, Resnet50, Xception, InceptionResNetV2, and MobileNet, on the dataset with and without repetition of images were compared. All the models were trained for 10 epochs with a batch size of 32. In every epoch training accuracy, training error, and validation accuracy, validation error was calculated. We adopted an Adaptive Momentum (Adam) optimizer with a learning rate (LR) of 0.001 and a loss function as a Categorical Cross Entropy. In order to make the optimizer converge faster and closer to the global minimum, an annealing method was used with a LR. To keep the advantage of the faster computation time with a high LR, we decreased the LR dynamically every four epochs, depending on the validation accuracy. With the ‘ReduceLROnPlateau’ function from ‘Keras.callbacks’, we chose to reduce the LR by half if the validation loss did not improve after four epochs.

Table 2 and Table 3 show the results of the transfer learning nets’ accuracy with and without repetition of images.

Table 3 clearly shows the difference produced when images were equalized in their frequencies. While some nets, such as VGG19, InceptionV3, and ResNet50, showed a decline in accuracy, there was an increase in accuracy for InceptionResNetV2, MobileNet, and Xception. The best performing net in the balanced dataset was Xception, while in unbalanced dataset it was MobileNet.

**Figure 4:**
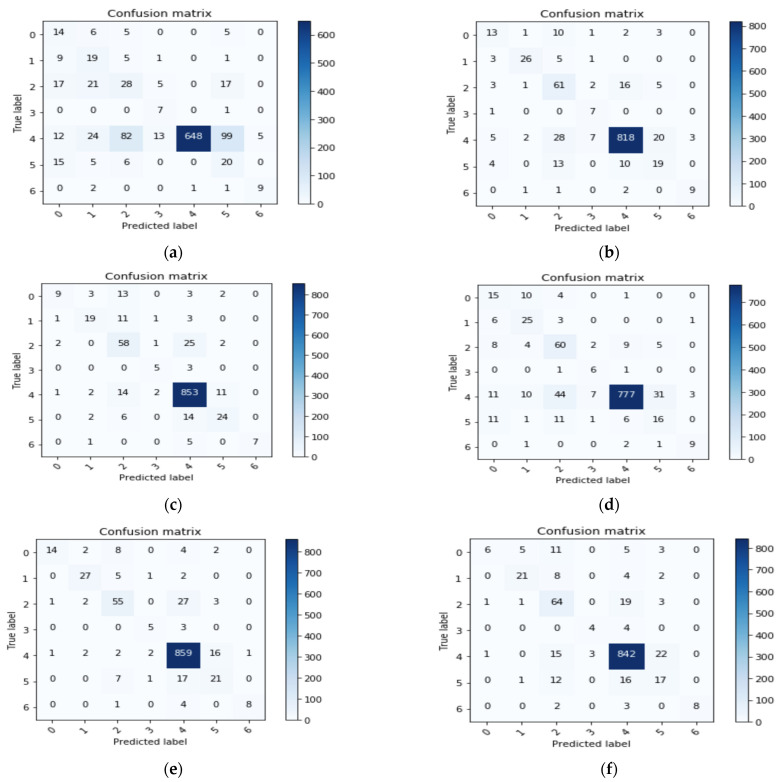
Confusion matrix results of different nets: (**a**) VGG19; (**b**) InceptionV3; (**c**) InceptionResNetV2; (**d**) ResNet50; (**e**) Xception; (**f**) MobileNet.

Here, we compared the performance of all seven nets by using a confusion matrix. A confusion matrix was constructed for every network. Performance of different nets was tested by passing 1002 randomly selected images (testing data). Accuracy was considered as a measure for calculating the performance with the skin lesion dataset. Figure 4a–f shows the confusion matrix results that we achieved for different nets.

From Figure 4, we can infer true positive (TP), true negative (TN), false positive (FP), and false negative (FN) values for each class. These values can help in calculating Precision, Recall, F1 Score, and accuracy.

**Figure 5:**
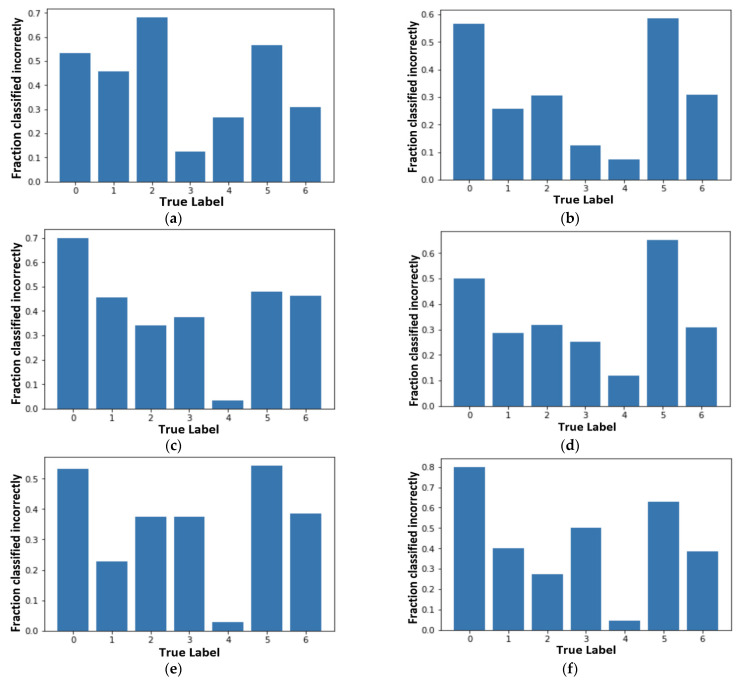
Fraction classified incorrectly for all seven models: (**a**) VGG19; (**b**) InceptionV3; (**c**) InceptionResNetV2; (**d**) ResNet50; (**e**) Xception; (**f**) MobileNet.

Incorrectly classified graphs in Figure 5 are derived from the confusion matrices. Figure 5 shows the incorrectly classified results for different nets. They can be calculated as the percentage of incorrect classification out of the total number of images in each class. We can follow the same process for correctly classified graphs. Figure 5a–f shows the fraction that has been classified incorrectly by the different transfer learning nets and our proposed model. From all the subfigures, it is evidently clear that ‘Akiec’ (label ‘0’) and ‘Mel’ (label ‘5’) are the most difficult lesions to be identified in the system. Additionally, ‘Nv’ (label ‘4’), having the least incorrect classification, was correctly classified by the system. In the VGG19 model, ‘Bkl’ (label ‘2’) performed worst out of all the remaining classes. MobileNet presented the highest classification accuracy for label ‘2’, with respect to models. ‘Df’ (label ‘3’) was easily identifiable by VGG19 and InceptionV3 compared to the rest, whereas ‘Nv’ (label ‘4’) showed better results in Inception-ResNet-v2, MobileNet, and XceptionNet. From the analysis, Figure 5e has most of the lowest incorrectly classified values and the highest accuracy.

**Figure 6:**
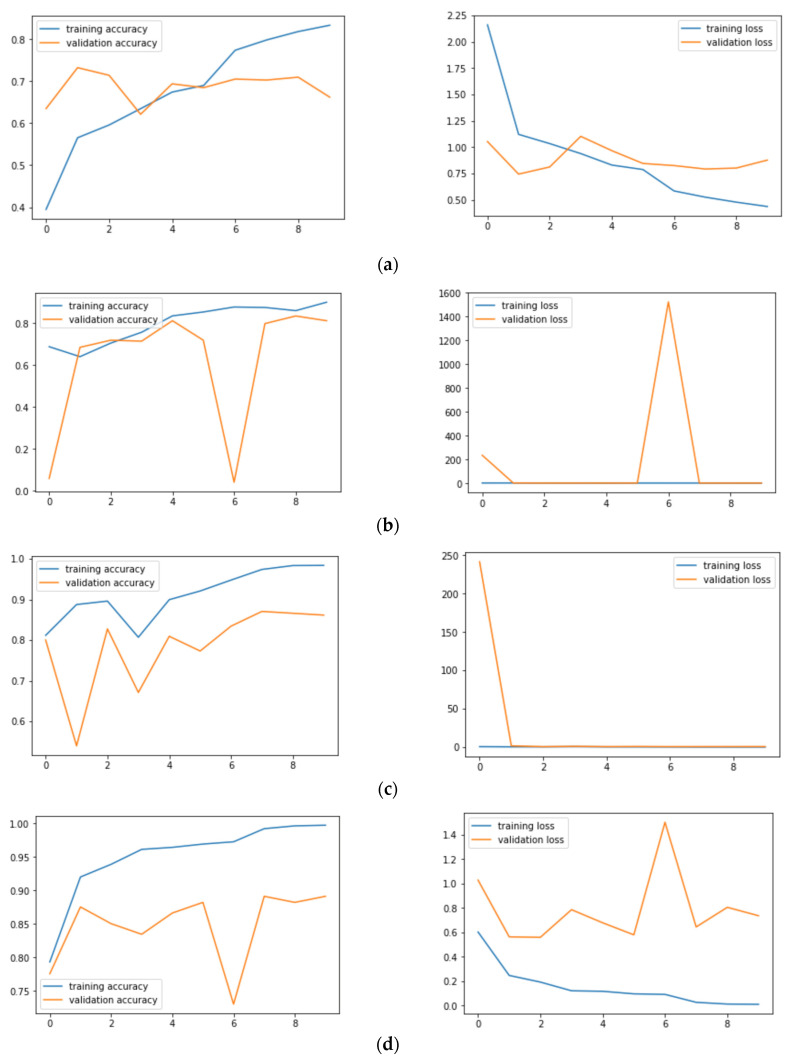

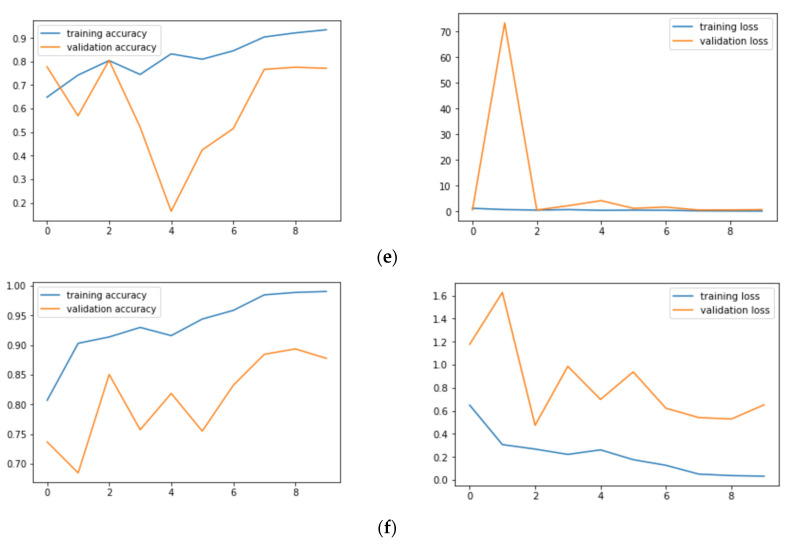
Training and validation accuracies and training and validation loss: (**a**) VGG19; (**b**) InceptionV3; (**c**) InceptionResNetV2; (**d**) ResNet50; (**e**) Xception; (**f**) MobileNet.

Figure 6a–f shows the training: validation accuracy and loss for all the six models. Figure 6a shows that VGG19 has a validation loss of 0.88 and an accuracy of 67.54% on skin lesion classification. Figure 6b shows that InceptionV3 has a validation loss of 0.66 and an accuracy of 86.40%, while Figure 6c shows that InceptionResNetV2 has a validation loss of 0.68 and an accuracy of 88.40%. Figure 6d shows that Xception has a validation loss of 0.58 and an accuracy of 89.66%, and Figure 6e shows that Resnet50 has a validation loss of 0.74 and an accuracy of 82.32%. Finally, Figure 6f shows that MobileNet has a validation loss of 0.65 and an accuracy of 87.21%. From the Figure 6a–f, we can also infer that Xception Net’s loss lies in the range of 0 to 2, thereby incurring minimum fluctuations.

(2) $$Precision=\frac{TP}{TP+FP},\;Recall=\frac{TP}{TP+FN}$$

(3) $$F1-Score=\frac{2*Precision*Recall}{Precision+Recall}$$

(4) $$Accuracy=\frac{TP+TN}{TP+TN+FP+FN}$$

where *TP*, *TN*, *FP*, and *FN* denote the true positives, true negatives, false positives, and false negatives, respectively. By making use of the above formulae, we can calculate precision, recall, and f-measure for each class and also get the information of which network performs best. In addition, the average of the recall, precision, and F-measure among classes will be computed in order to obtain a precise measure that it is not corrupted by the class imbalance. Note that the average recall is the equivalent to the balanced accuracy for multi-class problems. In the table below, we present the average values of precision, recall, F-measure, and accuracies.

From Table 4, it is clear that the Xception Net has the highest values in terms of accuracy, recall, precision, and F-Measure. Let’s take a look at the individual values of each class in the Xception Net to get a better understanding.

From the above Table 5, Label 4 (Nv) has the highest precision of 94% and Label 5 (Mel) has the lowest, with 50%. Label 6 (vasc), although having a lower number of images in the validation set, has proved to show a good precision, along with Label 0 (Akiec) and Label 1 (Bcc), respectively. Table 6 gives the results of accuracies and losses on our test set. We infered that the Xception net had the lowest loss of 0.5168 and the highest achieved accuracy of 90.48% in our work.

### Computational Cost

The computational costs of the simulations is provided here (Table 7 and Table 8) in the form of hardware used and the computational cost involved for different models.

## 4. Discussion and Conclusions

Due to the COVID-19 situation, everyone has suffered a lot but also gained a lot [49]. On one side, a large number of populations have contracted coronavirus, and many have died, but it is nowhere near to the upcoming UV radiations, which would have penetrated the ozone layer. Because of this pandemic situation and people staying in their homes, this has caused the ozone layer hole, which was getting bigger day by day, to close up. Skin cancers can be now diagnosed using these tools and can be treated earlier, and we can save more lives.

Through this research work, it is demonstrated that it is possible to achieve a competitive classification performance by using different types of data augmentation and transfer learning methods. Using the data augmentation method, we could get nearly 32k images, and we then performed feature extraction to get the required results. It is inferred that Xception Net outperforms the rest of the transfer learning nets used. It was observed that Label 0 (Akiec) and Label 5 (Mel) were most incorrectly classified because of their extreme resemblance to simple skin patches that are not harmful. Xception Net provides us with an accuracy of 90.48. It has the highest recall, precision, and F-Measure values, which are 89.57, 88.76, and 89.02 respectively. InceptionResNetV2 and MobileNet follow Xception Net closely in the prediction of results. Melanocytic Nevi is the most accurately classified skin lesion. There is a necessity of further and extensive research in this field as skin cancer-caused deaths are taking a toll. Transfer learning algorithms differ from those used in this paper, and proper fine-tuning may result in better accuracy.

## Figures and Tables

**Table 1:** Data augmentation of the dataset.

Disease	Frequency before Augmentation	Multiply Factor (k)	Frequency after Augmentation
Melanocytic Nevi	3179	1	3179
Benign Keratosis	317	10	3170
Melanoma	165	19	3135
Basal Cell Carcinoma	126	25	3150
Actinic Keratosis	109	29	3161
Vascular Skin Lesions	46	69	3174
Dermatofibroma	28	110	3080

**Table 2:** Performance of transfer learning nets without repetition of images.

Model without Repetition	Accuracy	Avg. Recall	Avg. Precision	Avg. F-Measure
VGG19	0.6718	0.67	0.78	0.71
InceptionV3	0.8168	0.82	0.75	0.78
InceptionResnetV2	0.8114	0.81	0.82	0.80
ResNet50	0.8105	0.81	0.75	0.77
Xception	0.8096	0.81	0.78	0.78
MobileNet	0.8241	0.82	0.84	0.80

**Table 3:** Performance of transfer learning nets with repetition of images.

Model with Repetition	Accuracy	Avg. Recall	Avg. Precision	Avg. F-Measure
VGG19	0.66	0.66	0.86	0.72
InceptionV3	0.79	0.79	0.87	0.82
InceptionResnetV2	0.85	0.86	0.88	0.86
ResNet50	0.77	0.78	0.86	0.80
Xception	0.90	0.90	0.90	0.90
MobileNet	0.88	0.89	0.88	0.88

**Table 4:** Precision, recall, F-Measure, and accuracy values of the models.

Model	Accuracy	Avg. Recall	Avg. Precision	Avg. F-Measure
VGG19	0.6754	0.6734	0.8548	0.7479
InceptionV3	0.8640	0.8619	0.8769	0.8713
InceptionResnetV2	0.8840	0.8762	0.8793	0.8845
ResNet50	0.8232	0.8222	0.8680	0.8416
Xception	**0.8966**	**0.8957**	**0.8876**	**0.8902**
MobileNet	0.8721	0.8711	0.8838	0.8740

**Table 5:** Xcpetion Net precision, recall, and F1-Score values.

Disease	Avg. Precision	Avg. Recall	Avg. F-Measure
Melanocytic Nevi	0.94	0.98	0.96
Benign Keratosis	0.68	0.68	0.68
Melanoma	0.58	0.48	0.52
Basal Cell Carcinoma	0.88	0.80	0.84
Actinic Keratosis	0.92	0.37	0.52
Vascular Skin Lesions	1.0	0.69	0.82
Dermatofibroma	0.71	0.62	0.67

**Table 6:** Test accuracy and loss values of all learning nets used.

Transfer Learning Nets	Accuracy	Loss
VGG19	66.36	1.0134
Resnet50	77.60	0.6855
InceptionResNetV2	85.58	0.6745
InceptionV3	79.23	0.6665
Xception	90.48	0.5168
MobileNet	88.57	0.6347

**Table 7:** Hardware specification.

Hardware Use	Specification
NVIDIA GPU	Tesla P100
CUDA Version	9.2
GPU RAM (GB)	17.1
CPU Chip	Intel Xeon CPU
Chip Speed (GHz)	2.2 or 2.3
CPU Cores	2
CPU RAM (Total GB)	16.4
L3 Cache (MB)	46
Disk Space (Total GB)	220

**Table 8:** Computation time.

Model Name	Computational Time (In Seconds)
VGG19	746.84069
InceptionV3	751.12284
InceptionResnetV2	2456.34356
ResNet50	761.63929
Xception	834.66028
MobileNet	695.36065

## Data Availability

Data is available in the manuscript.
